# Molecular linkage tracing of HIV-1 transmission events in seroconcordant couples in Guangxi Province, Southeastern China

**DOI:** 10.1186/s40064-016-3578-2

**Published:** 2016-11-25

**Authors:** Nidan Wang, Zhenzhu Tang, Yijia Li, Peiyan Xie, Yiming Shao

**Affiliations:** 1 State Key Laboratory for Infectious Disease Prevention and Control, National Center for AIDS/STD Control and Prevention, Chinese Center for Disease Control and Prevention, Beijing, People’s Republic of China; 2 Guangxi Center for Disease Prevention and Control, Nanning, People’s Republic of China; 3 Department of Infectious Diseases, Peking Union Medical College Hospital, Beijing, People’s Republic of China; 4 Collaborative Innovation Center for Diagnosis and Treatment of Infectious Diseases, Hangzhou, People’s Republic of China

**Keywords:** Serodiscordant couples, Seroconcordant couples, HIV-1 transmission, Phylogenetic analysis, Bayesian analysis

## Abstract

**Background:**

Guangxi Province in Southeastern China has one of the highest HIV-1 infection and transmission rates in stable couples. However, the mode of transmission at the molecular level has seldom been reported amongst this group. It is important to investigate this issue to support the treatment-as-prevention approach and for efficient interventions.

**Methods:**

HIV-1 subgenomic regions (1.2 kb of *pol* and a 660-bp *env* C2V5 fragment) were sequenced in 42 couples. A couple linkage assessment was performed by phylogenetic analysis of sequences and Bayesian analysis of genetic distances. A subset of pairs was selected for single-genome amplification.

**Results:**

Thirty-five pairs (83.3 %, 35/42) were identified as linked, 3 pairs (7.1 %, 3/42) were identified as indeterminate, and 4 pairs (9.5 %) were identified as unlinked. The predominant intra-couple-transmitted HIV-1 subtype was CRF01_AE (80 %, 28/35). The median genetic distance of linked couples was 0.5 %.

**Conclusion:**

The majority of HIV-1 transmission events in this study occurred within the partnership, and the predominant HIV-1 subtype was CRF01_AE. Further research on the mode of HIV transmission in other locations is needed.

**Electronic supplementary material:**

The online version of this article (doi:10.1186/s40064-016-3578-2) contains supplementary material, which is available to authorized users.

## Background

Guangxi Province in China has a particularly high prevalence of HIV-1 infection. The complexity of the circulating viral subtypes and the high frequency of infection events in this region have placed a considerable economic burden on the government and posed great challenges to the HIV-1 disease control systems (Feng et al. 2013; Zeng et al. 2012). According to national and local molecular epidemiology reports, commercial heterosexual intercourse and intravenous drug use are two major routes of HIV-1 transmission in Guangxi. For infected people, their seronegative stable sexual partners are at a high risk of infection (He et al. 2012; Li et al. 2013). Two retrospective observational cohort studies performed in China showed that anti-retroviral therapy can reduce the seroconversion rate of HIV-1 seronegative partners; however, no molecular analysis was presented in these reports (Jia et al. 2012; Tang et al. 2015). Therefore, the possibility that seroconverters may have been infected from outside of the stable sexual partnership cannot be excluded. Our study aimed to investigate the mode of HIV transmission in heterosexual couples living in Guangxi using molecular methods to determine the optimum treatment programmes.

## Methods

To improve the reliability of our results, both the HIV *pol* and *env* genes were analysed in this study (English et al. 2011; Sturmer et al. 2004). The phylogenetic linkage of HIV viruses from couples in Guangxi was analysed using genetic distance and Bayesian methods. The final results from linkage analysis were confirmed using single-genome amplification (SGA) on a subset of the population examined (Boily et al. 2010; Eyer-Silva and Morgado 2006; Jennes et al. 2012).

### Sample sources and data collection

From July 2006 until April 2013, participants at voluntary counselling and testing (VCT) centres in Guangxi that tested positive for HIV-1 during screening donated plasma and whole blood samples for routine HIV-1 diagnostic assays. If they had stable partners, the individuals and their seronegative cohabitating partners had follow-up interviews and were tested at the same time. If the negative partners experienced seroconversion during the follow-up, HIV diagnostic assays and epidemiological data were collected into a serodiscordant database (Table 1, Jia et al. 2012). If the partners were already seropositive at the first follow-up, then the possible transmission time and direction were inferred by separate questionnaires, and these couples were entered into a seroconcordant database (Table 2). In this study, plasma samples obtained from routine deposits at the Guangxi Center for Disease control (CDC) were screened and selected.

**Table 1:** Conclusion of HIV-1 transmission linkage for 20 sero discordant couples and transmission direction

Couple number	Donor				Recipient				Conclusion
	First test^a^	First CD4 count	Suspect HIV source^b^	HIV subtype	First test^a^	First CD4 count	Suspect HIV source^b^	HIV subtype	
									Transmission direction
Linked (n = 20)									
024	2008.7	N/A^c^	Extramarital sex	01AE	2011.11	348	Extramarital sex	01AE	Wife to husband
027	2010.6	81	Extramarital sex	01AE	2010.9	N/A	Husband	01AE	Husband to wife
034	2011.11	420	Extramarital sex	08BC	2012.3	495	Husband	08BC	Husband to wife
046	2011.1	21	Extramarital sex	01AE	2011.3	N/A	Husband	01AE	Husband to wife
048	2010.2	229	Extramarital sex	07BC	2010.11	N/A	Wife	07BC	Wife to husband
050	2008.7	8	Extramarital sex	01AE	2008.11	218	Husband	01AE	Husband to wife
054	2011.12	383	Extramarital sex	01AE	2012.3	777	Husband	01AE	Husband to wife
057	2010.9	397	Drug use	08BC	2011.3	N/A	Husband	08BC	Husband to wife
117	2011.1	94	Blood transfus	01AE	2011.7	676	Husband	01AE	Husband to wife
128	2011.5	36	Extramarital sex	01AE	2011.11	273	Husband	01AE	Husband to wife
168	2010.11	26	Extramarital sex	01AE	2011.7	432	Husband	01AE	Husband to wife
169	2010.12	23	Extramarital sex	01AE	2012.9	N/A	N/A	01AE	Husband to wife
223	2009.12	524	Extramarital sex	01AE	2011.8	449	Husband	01AE	Husband to wife
245	2010.9	605	Extramarital sex	01AE	2010.12	N/A	Husband	01AE	Husband to wife
252	2011.1	462	Extramarital sex	01AE	2011.6	N/A	Wife	01AE	Wife to husband
254	2011.1	310	Extramarital sex	08BC	2012.6	458	Husband	08BC	Husband to wife
259	2011.3	511	Extramarital sex	01AE	2011.7	N/A	Husband	01AE	Husband to wife
276	2008.6	N/A	Extramarital sex	01AE	2011.6	N/A	Wife	01AE	Wife to husband
278	2011.6	N/A	Extramarital sex	08BC	2012.3	352	Extramarital sex	08BC	Husband to wife
300^*d*^	2010.6	629	Extramarital sex	01AE	2013.4	N/A	Wife	01AE	Wife to husband

^a^The time of the first sero-positive date

^b^The transmission route collected from the questionnaire

^c^N/A, not available

^d^Defined by epidemiology data

**Table 2:** Conclusion of HIV-1 transmission linkage for 22 sero concordant couples

Couple number	Donor				Recipient			
	First test^a^	First CD4 count	Suspect HIV source^b^	HIV subtype	First test^a^	First CD4 count	Suspect HIV source^b^	HIV subtype
Linked (n = 15)								
283	2003.11	353	Drug use	01AE	N/A	N/A	Husband	01AE
284	N/A^d^	N/A	N/A	08BC	N/A	N/A	N/A	08BC
286	2010.5	169	Extramarital sex	01AE	2010.12	449	Husband	01AE
287	2010.6	444	Extramarital sex	01AE	2010.6	434	Husband	01AE
289	2010.11	N/A	Extramarital sex	07BC	2010.11	491	Husband	07BC
291	2012.10	129	Extramarital sex	01AE	2013.1	268	wife	01AE
292	2012.10	N/A	Extramarital sex	01AE	2012.10	280	Husband	01AE
295	2013.2	22	Extramarital sex	01AE	2013.2	197	Husband	01AE
296	2013.1	401	Extramarital sex	01AE	2013.2	286	Husband	01AE
297	2012.3	101	Extramarital sex	01AE	2012.3	234	Husband	01AE
298	2012.1	408	Extramarital sex	01AE	2012.1	327	Extramarital sex	01AE
299	2012.11	20	Extramarital sex	01AE/BC	2012.11	467	Husband	01AE/BC
304	2011.11	369	Extramarital sex	01AE	2011.12	N/A	Husband	01AE
305	2013.4	381	N/A	01AE	2013.4	264	N/A	01AE
306	2013.4	316	Extramarital sex	01AE	2013.4	443	Extramarital sex	01AE
Indeterminate (n = 3)								
290	2010.1	N/A	Extramarital sex	01AE	2010.1	N/A	Husband	01AE
293	2012.9	11	Extramarital sex	01AE	2012.9	115	Husband	01AE
307	2012.11	22	Extramarital sex	01AE	N/A	N/A	N/A	01AE
Unlinked (n = 4)								
282	2006.7	505	Drug use	01AE	2002.8	N/A	N/A	01AE
288	2013.2	331	Extramarital sex	01AE	2009.6	71	Extramarital sex	07BC
294	2010.6	611	N/A	08BC	2012.7	373	Extramarital sex	08BC
309	N/A	N/A	N/A	01AE	N/A	N/A	N/A	01AE

^a^The time of the first sero-positive date

^b^The transmission route collected from the questionnaire

^c^N/A, not available

^d^Defined by epidemiology data

The study was approved by the Institutional Review Board of the National Center for AIDS/STD Control and Prevention. Informed consent was obtained from all participants at the time of sample collection.

### Viral RNA (vRNA) isolation, amplification and sequencing

Total viral RNA was extracted from 280 µl of thawed plasma using the Qiagen Viral RNA Mini Kit according to the manufacturer’s instructions (Qiagen, Germany). The fragment spanning the HIV-1 *pol* gene region (1.2 kb nucleotides in length), encoding all 99 amino acids in HIV protease and the first 317 amino acids in HIV reverse transcriptase, was amplified by one-step reverse transcription polymerase chain reaction (RT-PCR) (Phan et al. 2015). Considering the relatively high diversity of the envelope region, C2V5 sequences (HXB2 coordinates: 7002–7663) were amplified using near-limiting-dilution PCR. PCR products were purified and directly sequenced to keep the ambiguous base rate below 0.3 %. Technicians were blinded to specimen partnerships and conducted analyses on the index and partner samples in different rooms to minimize the risk of specimen contamination (Etemad et al. 2015). Additional details regarding the laboratory methods have been described in a previous report (Campbell et al. 2011).

### Genotyping HIV-1 sequences by phylogenetic tree analysis

All *pol* and *env* sequences were aligned with HIV-1 reference subtypes (A–D, F–H, J, and K) obtained from the Los Alamos HIV Database (http://hiv-web.lanl.gov/) using MUSCLE in MEGA 5 software. Aligned columns with more than 50 % gaps were deleted by Gapstrip. The phylogenetic model was determined using the best-fit model estimated by FindModel (http://hiv-web.lanl.gov/). Maximum-likelihood trees were constructed with MEGA 5 software (Tamura et al. 2011) and bootstrap values were calculated from 500 replicates.

### Phylogenetic linkage analysis of couple sequences

Maximum-likelihood trees of *pol* and *env* sequences were constructed using a general time reversible (GTR) nucleotide substitution with a gamma distribution of rates. To assure that the cluster represented the real relationship as much as possible, unrelated sequences from a local area (53 pol seq and 54 env seq) were added as controls and additional criteria were utilized, as reported in a previous study (Trask et al. 2002). We considered cluster bootstrap values ≥80 % to be indicative of genetic linkage and bootstrap values <80 % to be indeterminate, which were further analysed by pairwise genetic distance; we considered sequences separated in different branches as genetically unlinked (Jennes et al. 2012). According to our criteria, a couple with either of the two HIV genes (cluster bootstrap value >80 %) was determined to have a linked transmission relationship. The corresponding pairwise genetic distances of couples were calculated separately using MEGA 5 software, with a transition/transversion ratio of 0.5.

### Bayesian analysis of genetic distances

We used a Bayesian algorithm to derive an estimate of the probability of the linkage between sequences in the cohort by pairwise genetic distance distribution, as previously reported. According to kernel density estimation, we calculated conditional densities of the genetic distances [f(X|linked)] from couples’ sequences in the serodiscordant cohort, which are epidemiologically and phylogenetically linked (linked training). Using the same method, we calculated f(X|unlinked) from single partner sequences in the control dataset (unlinked training). An empirical Bayes’ approach was used, and the initial value for P (linked) was 0.5; the posterior probabilities of linkage for the seroconcordant cohort was based on the linked and unlinked training data (more details in Additional file 1: Figure S1). The posterior probability of the linkage for each sequence pair, i, is given by Bayes’ formula:

$$ P\left( {linked |X_{i} } \right) = \frac{{P (linked) * f(linked|X_{i} )}}{{P (linked) * f(linked|X_{i} ) + (1 - P \left( {linked} \right))* f(unlinked|X_{i} )}} $$

### Analysis of HIV *env* by single-genome amplification

Single-genome amplification was performed in five undetermined or unlinked couples to investigate the linkage relationship between the viral quasispecies of the partners to provide additional evidence to support our results (Haaland et al. 2009; Salazar-Gonzalez et al. 2009). vRNA was extracted and reverse-transcribed to generate cDNA, which was diluted to an optimum concentration to guarantee less than 20 C2V5 env gene PCR products in one reaction well (Salazar-Gonzalez et al. 2009). Phylogenetic trees of each couple were generated by the maximum-likelihood method using the SGA sequences to investigate the topology of paraphyly of the transmitted source (Scaduto et al. 2010).

## Results

### Subject characteristics and sequence amplification

For this study, we collected 154 frozen serum samples from the Guangxi CDC’s couple library. Additional details on the subject demographic characteristics are provided in Additional file 2: Table S1. Amongst these samples, there were 50 pair samples and 54 single partner samples. Serodiscordant couple samples were collected around the time of seroconversion. Seroconcordant couple samples were selected at the first time of the index partners’ diagnosis. The sample set represented several geographically distinct areas of Guangxi.

Forty-one *pol* pair sequences had both partners’ samples, including 20 couples from the serodiscordant cohort and 21 couples from the seroconcordant cohort (Additional file 2: Table S1). Thirty-two *env* pair sequences were obtained as follows: 11 were from serodiscordant couples and 21 were from seroconcordant couples. Another 53 *pol* and 54 *env* sequences were obtained from a couple’s cohort in Guangxi Province, but their partners’ samples were not supplied or they experienced amplification failure; therefore, these samples were used as controls to represent the local circulating virus.

### The CRF01_AE subtype highly correlates with most couples in Guangxi

**Fig. 1:**
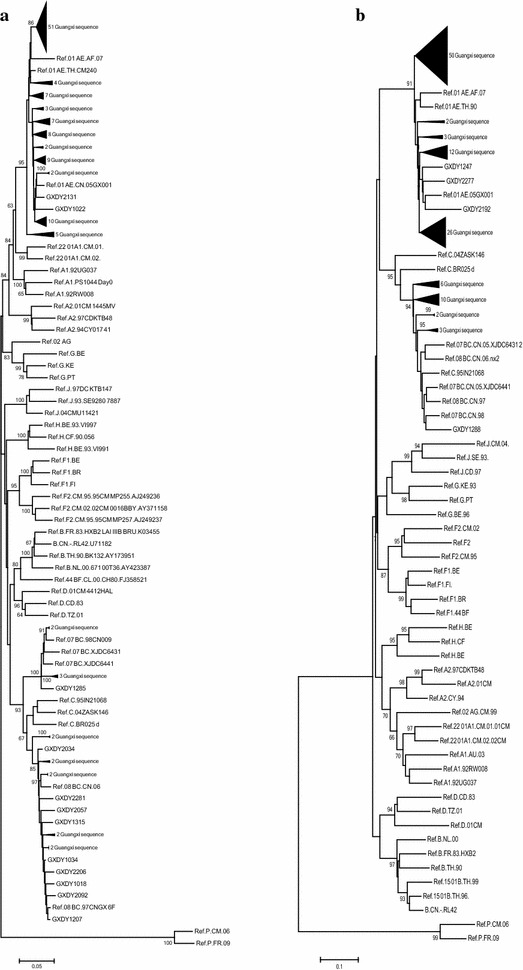
The transmission cases involved subtype analysis. Maximum likelihood trees were created with *pol* (**a**) and *env* C2V5 (**b**) gene regions of HIV-1 sequences from couples, unrelated individuals from local areas, and a selection of reference sequences of subtype A–D, F–H, J, K, CRF01_AE, CRF07_BC, and CRF08_BC (http://hiv-web.lanl.gov/)

A total of 118 *env* sequences and 135 *pol* sequences were obtained from this survey. According to the genotype results from the HIV database reference assignment, the major HIV-1 subtype was CRF01_AE (81.5 %, 110/135), followed by subtypes CRF08_BC (12.6 %, 17/135), CRF07_BC (4.4 %, 6/135) and URFs (1.5 %, 2/135) (Fig. 1). This is consistent with a multicentre molecular epidemiological observation in which subtype CRF01_AE was predominant in HIV patients infected via sexual transmission (Li et al. 2013).

### Most HIV infections in surveyed couples are genetically linked

**Fig. 2:**
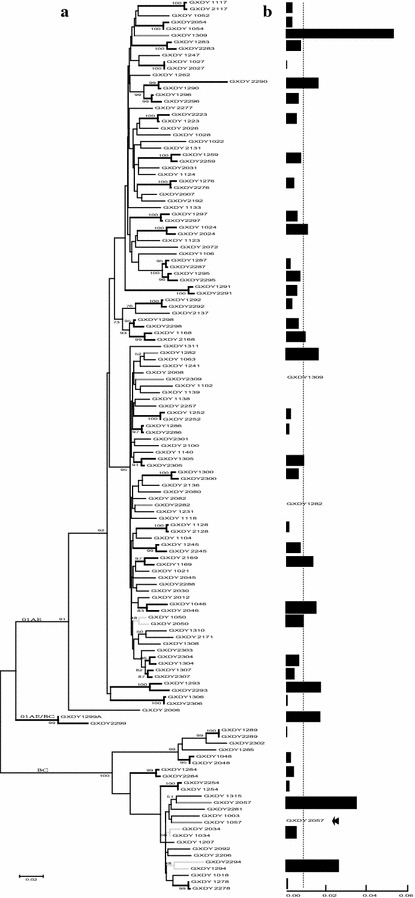
Molecular analysis of HIV-1 transmission in 41 couples by *pol* gene. **a** Maximum-likelihood phylogenetic tree. The first digit of the sequence numbers differentiates between the partners: “*1*” indicates the donor, “*2*” indicates the recipient; the remaining three digits represent the couple’s ID. Bootstrap values are shown for the grouping of couple sequences for each event, based on 1000 bootstrap replicates. **b** Maximum-likelihood genetic distance. The *color and site of bars* correspond to couples diagramed on the *left*. For the non-clustered couples, only one sequence has the *bar value* and the other sequence is labeled with the partner’s ID. The *dotted line* represents the median pairwise distance of the linked pairs

Genetic linkage was evaluated through phylogenetic tree analysis on the conserved *pol* and highly variable *env* C2V5 viral genes in each couple. A total of 41 *pol* sequence pairs and 32 *env* sequence pairs were obtained. Amongst these, 31 couples had both *pol* and *env* sequence pairs. Sequence pairs from 30 of 33 couples infected with CRF01_AE were very closely related and clustered in exclusive branches with high bootstrap values in the *pol* phylogenetic trees (Fig. 2). The median pairwise distance of these couples in the *pol* region was 0.008, indicating that they share a closely related CRF01_AE viral ancestor. Sequence pairs from two couples infected with CRF07_BC and three couples infected with CRF08_BC that clustered with high bootstrap values were defined as linked. Sequences from two other couples (GXDY1034-2034 and GXDY1294-2294) that clustered with low bootstrap values and one couple (GXDY1057-2057) with a pair sequence that was separated into a different branch were defined as unlinked (for details see Additional file 2: Table S1).

**Fig. 3:**
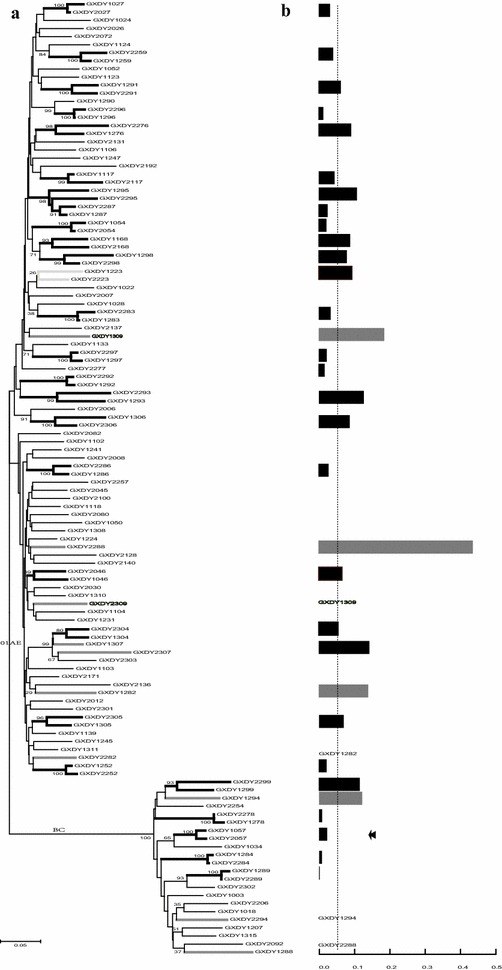
Molecular analysis of HIV-1 transmission in 32 couples by *env* gene. **a** Maximum-likelihood *env* phylogenetic tree. **b** Maximum-likelihood genetic distance. The same annotations apply here as for the *pol* gene in Fig. 2

For the *env* region, 21 CRF01_AE couples and five CRF_07BC/08BC couples were clustered with high bootstrap values, including another linked couple (GXDY1057-2057) that lacked *pol* sequences. The median pairwise distance of 21 CRF01_AE couples in the *env* region was 0.05 (Fig. 3).

In conclusion, 35 pairs (85.3 %, 35/41) in the *pol* phylogenetic tree and 26 pairs (81 %, 26/32) in the *env* phylogenetic tree were defined as linked by monophyly. Thirty-one pairs had both gene sequences. Amongst them, there were 25 pairs (80 %, 25/31) with linkage in both genes and four pairs were separated phylogenetically (GXDY1057-2057, GXDY1223-2223, GXDY1294-2294, and GXDY1307-2307).

### Genetic distance and Bayesian analyses

The median pairwise genetic distance was 0.5 % (range 0.0–4.1 %) in *pol* and 4.0 % (range 0.0–11.7 %) in *env*. In unlinked and indeterminate pairs, the genetic distance was 2.0 % (range 0.4–5.0 %) and 12 % (range 8–16 %) in *pol* and *env*, respectively. Median Bayesian posterior probabilities for linked (n = 17) and unlinked (n = 6) pairs in seroconcordant couples (n = 23 pairs) were 100.0 and 0.0 % in *pol* and 99.8 and 9.0 % in *env* (Additional file 2: Table S1), respectively.

In one couple (GXDY 1307–2307), the sequence pair was monophyletic, and posterior probabilities were high for pol (100.0 %) but low for *env* (9 %). The results from the phylogenetic and Bayesian analyses were inconsistent in three paired couples (GXDY1290–2290, GXDY1293–2293 and GXDY1294–2294) with respect to *pol* and one pair (GXDY1293–2293) with respect to *env*.

### Paraphyly was used to identify linked couples in the phylogenetic tree

We evaluated single env sequences in five pairs showing inconsistencies either between the two genes or between the two analytical methods used (GXDY1057–2057, GXDY1223–2223, GXDY1293–2293, GXDY1294–2294, and GXDY1307–2307). One pair (GXDY1290–2290) was classified as indeterminate for a lack of env SGA sequences.

**Fig. 4:**
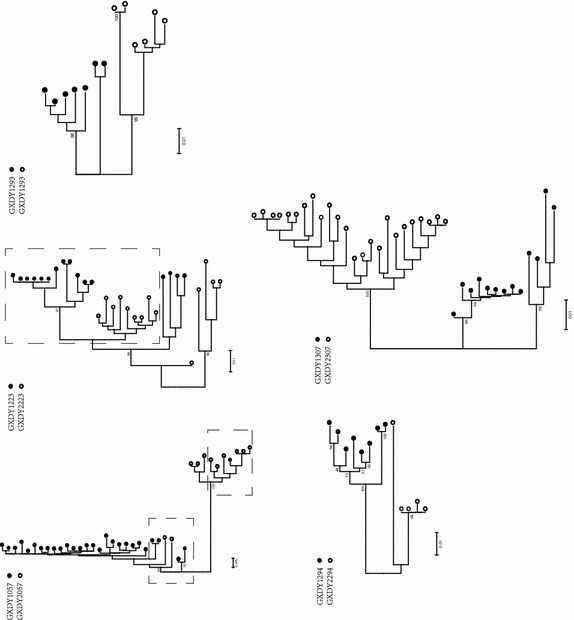
Single genome amplification analysis for five controversy couples. Aligned SGA sequences from each couple were used to generate maximum-likelihood phylogenetic trees independently based on 1000 bootstrap replicates. Here the pairs no. 057, 223, 293, 294, and 307 are analyzed. *Black circle* indicates the donor, *white circle* indicates the recipient

Results from the linked couples showed that sequences from the donors were paraphyletic with respect to the recipients’ sequences, as expected (Scaduto et al. 2010). For example, in the couple GXDY1223–2223, the GXDY2223 sequences were clustered with a small proportion of GXDY1223 sequences as monophyly (Fig. 4). This phenomenon suggests that the genetic distance of some quasispecies sequences from the donor and recipient are close enough to mingle with each other.

## Discussion

To accomplish the 2030 UN goal to end the HIV epidemic, new HIV-1 transmission should be controlled and the potential transmission source identified, especially in stable couples (Cousins 2016). In the famous HPTN 052 prospective research, robust evidence proved the efficiency of the treatment-as-prevention strategy (TAPS) after removing outside transmission couples (Eshleman et al. 2011). The sustained decrease in genetically-linked transmission in couples has been further proven in a newly published HPTN052 report (Cohen et al. 2016). Considering the huge population in China, the TASP approach has great significance in public health. A national retrospective analysis using epidemic data supports the HPTN052 conclusion (Jia et al. 2012), which is the same conclusion made in a recent study in Henan Province (Smith et al. 2015). A recent epidemiological investigation of serodiscordance in Guangxi again supported the efficiency of TASP. However, not one report has investigated the local transmission relationship at the molecular level in China. Here, we provide molecular evidence to complement the related research.

Phylogenetic analyses of suspected transmission relationships have been widely used to provide evidence and clues for many HIV-1 transmission events (Blanchard et al. 1998; Kim et al. 1999; Leitner et al. 1996; Lopes et al. 2015). Unlike previous reports of seroconcordant couples (Jennes et al. 2012), Guangxi Province has the following characteristics that render the identification of the viral transmission relationship challenging (Feng et al. 2013): an influx of a large number of individuals infected with different CRF01_AE lineages, a high rate of HIV-1 infection amongst drug users, and cross-infection amongst different risk groups that provides opportunities for viral recombination. This can be shown from the scale bar presenting the *pol* phylogenetic tree and one couple infected by a unique recombinant form (pair no. 299) (Wang et al. 2013). In our study, to identify the true relationship of these couples, phylogenetic analysis of two subgenomic regions combined with epidemiological data were used to determine the linkage status. Some pair sequences were further analysed phylogenetically using SGA to validate the results.

We identified 83.3 % (35/42) of intra-couple infections in our sample that were linked and 9.5 % (4/42) that were unlinked. The majority of cases were classified using phylogenetic analysis by bulk PCR. However, for the special cases in which the non-dominant quasispecies of the donor were transmitted or the viral variants of the recipient evolved with selection, the inference made by one or two sequences from couples could not reveal the true relationship. For example, the *pol* gene of pair no. 057 indicated unlinked transmission, which was inconsistent with the *env* gene results. From the SGA characterisation of the couple’s quasispecies, we found two distinct groups of progeny virus in the donor, and only the non-dominant variant was transmitted to his partner. Thus, viral diversity may complicate linkage analysis if the sequences chosen from the index and partner belong to two different dominant viruses (Sturmer et al. 2004).

Although there are many epidemiological studies on serodiscordant and seroconcordant couples in China, they are all based on the assumption that intra-couple transmissions are linked. For the first time, this cross-sectional study demonstrates the true relationship of the serodiscordant and seroconcordant couples in the local area using molecular techniques. In this study, we used the standard combination method to investigate the viral relationship of the couples and to learn about the genetic distance distribution of linked pairs.

In comparison with studies from other countries, all the data in this survey showed a comparable intra-family transmission rate in Guangxi, and the main HIV-1 infection source was extramarital occasional sex (Eshleman et al. 2011; Jennes et al. 2012; Trask et al. 2002). The predominant HIV-1 subtype in our sample set was CRF01_AE, which is mainly transmitted through sexual routes and has the characteristics of faster clinical progression than other subtypes (Li et al. 2014). All of these factors support the importance to initiate ART early as a preventative method in Guangxi serodiscordant couples to reduce intra-family sexual transmission.

There are several limitations to this study. First, the sample size is small and spans a long period of time. In future studies, we will enrol more participants from multiple centres. Secondly, the transmission direction of seroconcordant couples who lacked epidemic data were obtained from questionnaires; in future studies, proper mathematic models should be used to identify the direction (Yang et al. 2012). Lastly, this is a small-scale study using convenient samples. Future studies with larger sample sizes are warranted.

In conclusion, this is a comprehensive analysis that aimed to identify the mode of intra-family HIV transmission. The data presented here provide evidence of a transmission source. Further research into the serodiscordant and seroconcordant couples in Guangxi and other regions in China will provide more information about intra-family HIV transmission and will aid in the design of related intervention policies in this region.

## Sequence data

Nucleotide sequences of GXDY *pol* and GXDY *env* regions have been submitted to GenBank under accession numbers KF835116–KF835250 and KF834998–KF835115.

## Additional files

**Additional file 1: Figure S1.** Flow chart depicting the process of screening our sample set and 339 determining genetic linkage and direction of transmission.

**Additional file 2: Table S1.** Epidemiological analysis and conclusion of HIV-1 transmission linkage for 42 HIV-1 couples.

## References

[CR1] Blanchard A, Ferris S, Chamaret S, Guetard D, Montagnier L (1998). Molecular evidence for nosocomial transmission of human immunodeficiency virus from a surgeon to one of his patients. J Virol.

[CR2] Boily MC, Buve A, Baggaley RF (2010). HIV transmission in serodiscordant heterosexual couples. BMJ.

[CR3] Campbell MS, Mullins JI, Hughes JP, Celum C, Wong KG, Raugi DN, Sorensen S, Stoddard JN, Zhao H, Deng W, Kahle E, Panteleeff D, Baeten JM, McCutchan FE, Albert J, Leitner T, Wald A, Corey L, Lingappa JR (2011). Viral linkage in HIV-1 seroconverters and their partners in an HIV-1 prevention clinical trial. PLoS ONE.

[CR4] Cohen MS, Chen YQ, McCauley M, Gamble T, Hosseinipour MC, Kumarasamy N, Hakim JG, Kumwenda J, Grinsztejn B, Pilotto JH, Godbole SV, Chariyalertsak S, Santos BR, Mayer KH, Hoffman IF, Eshleman SH, Piwowar-Manning E, Cottle L, Zhang XC, Makhema J, Mills LA, Panchia R, Faesen S, Eron J, Gallant J, Havlir D, Swindells S, Elharrar V, Burns D, Taha TE, Nielsen-Saines K, Celentano DD, Essex M, Hudelson SE, Redd AD, Fleming TR (2016). Antiretroviral therapy for the prevention of HIV-1 transmission. N Eng J Med..

[CR5] Cousins S (2016). Progress towards 2030 UN goal to end HIV epidemic falters. BMJ.

[CR6] English S, Katzourakis A, Bonsall D, Flanagan P, Duda A, Fidler S, Weber J, McClure M, Phillips R, Frater J (2011). Phylogenetic analysis consistent with a clinical history of sexual transmission of HIV-1 from a single donor reveals transmission of highly distinct variants. Retrovirology.

[CR7] Eshleman SH, Hudelson SE, Redd AD, Wang L, Debes R, Chen YQ, Martens CA, Ricklefs SM, Selig EJ, Porcella SF, Munshaw S, Ray SC, Piwowar-Manning E, McCauley M, Hosseinipour MC, Kumwenda J, Hakim JG, Chariyalertsak S, de Bruyn G, Grinsztejn B, Kumarasamy N, Makhema J, Mayer KH, Pilotto J, Santos BR, Quinn TC, Cohen MS, Hughes JP (2011). Analysis of genetic linkage of HIV from couples enrolled in the HIV prevention trials network 052 trial. J Infect Dis.

[CR8] Etemad B, Ghulam-Smith M, Gonzalez O, White LF, Sagar M (2015). Single genome amplification and standard bulk PCR yield HIV-1 envelope products with similar genotypic and phenotypic characteristics. J Virol Methods.

[CR9] Eyer-Silva WA, Morgado MG (2006). Molecular epidemiology of HIV-1 infection in a small Brazilian county: usefulness of envelope and polymerase sequences to epidemiologic studies. J Acquir Immune Defic Syndr.

[CR10] Feng Y, He X, Hsi JH, Li F, Li X, Wang Q, Ruan Y, Xing H, Lam TT, Pybus OG, Takebe Y, Shao Y (2013). The rapidly expanding CRF01_AE epidemic in China is driven by multiple lineages of HIV-1 viruses introduced in the 1990s. AIDS.

[CR11] Haaland RE, Hawkins PA, Salazar-Gonzalez J, Johnson A, Tichacek A, Karita E, Manigart O, Mulenga J, Keele BF, Shaw GM, Hahn BH, Allen SA, Derdeyn CA, Hunter E (2009). Inflammatory genital infections mitigate a severe genetic bottleneck in heterosexual transmission of subtype A and C HIV-1. PLoS Pathog.

[CR12] He X, Xing H, Ruan Y, Hong K, Cheng C, Hu Y, Xin R, Wei J, Feng Y, Hsi JH, Takebe Y, Shao Y (2012). A comprehensive mapping of HIV-1 genotypes in various risk groups and regions across China based on a nationwide molecular epidemiologic survey. PLoS ONE.

[CR13] Jennes W, Kyongo JK, Vanhommerig E, Camara M, Coppens S, Seydi M, Mboup S, Heyndrickx L, Kestens L (2012). Molecular epidemiology of HIV-1 transmission in a cohort of HIV-1 concordant heterosexual couples from Dakar. Senegal. PLos ONE.

[CR14] Jia Z, Ruan Y, Li Q, Xie P, Li P, Wang X, Chen RY, Shao Y (2012). Antiretroviral therapy to prevent HIV transmission in serodiscordant couples in China (2003–2011): a national observational cohort study. Lancet.

[CR15] Kim YB, Cho YK, Lee HJ, Kim CK, Kim YK, Yang JM (1999). Molecular phylogenetic analysis of human immunodeficiency virus type 1 strains obtained from Korean patients: env gene sequences. AIDS Res Hum Retroviruses.

[CR16] Leitner T, Escanilla D, Franzen C, Uhlen M, Albert J (1996). Accurate reconstruction of a known HIV-1 transmission history by phylogenetic tree analysis. Proc Natl Acad Sci USA.

[CR17] Li L, Chen L, Liang S, Liu W, Li T, Liu Y, Li H, Bao Z, Wang X, Li J (2013). Subtype CRF01_AE dominate the sexually transmitted human immunodeficiency virus type 1 epidemic in Guangxi, China. J Med Virol.

[CR18] Li Y, Han Y, Xie J, Gu L, Li W, Wang H, Lv W, Song X, Routy JP, Ishida T, Iwamoto A, Li T (2014). CRF01_AE subtype is associated with X4 tropism and fast HIV progression in Chinese patients infected through sexual transmission. AIDS.

[CR19] Lopes GI, Coelho LP, Hornke L, Volpato AP, Lopercio AP, Cabral GB, Ferreira JL, Domingues CS, Brigido LF (2015). Transmission of a multidrug-resistant HIV-1 from an occupational exposure, in Sao Paulo, Brazil. AIDS.

[CR20] Phan CT, Pham HV, Bi X, Ishizaki A, Saina M, Phung CD, Khu DT, Ichimura H (2015). Genetic Analyses of HIV-1 Strains transmitted from mother to child in Northern Vietnam. AIDS Res Hum Retroviruses.

[CR21] Salazar-Gonzalez JF, Salazar MG, Keele BF, Learn GH, Giorgi EE, Li H, Decker JM, Wang S, Baalwa J, Kraus MH, Parrish NF, Shaw KS, Guffey MB, Bar KJ, Davis KL, Ochsenbauer-Jambor C, Kappes JC, Saag MS, Cohen MS, Mulenga J, Derdeyn CA, Allen S, Hunter E, Markowitz M, Hraber P, Perelson AS, Bhattacharya T, Haynes BF, Korber BT, Hahn BH, Shaw GM (2009). Genetic identity, biological phenotype, and evolutionary pathways of transmitted/founder viruses in acute and early HIV-1 infection. J Exp Med.

[CR22] Scaduto DI, Brown JM, Haaland WC, Zwickl DJ, Hillis DM, Metzker ML (2010). Source identification in two criminal cases using phylogenetic analysis of HIV-1 DNA sequences. Proc Natl Acad Sci USA.

[CR23] Smith MK, Westreich D, Liu H, Zhu L, Wang L, He W, Zhou J, Miller WC, Cohen MS, Wang N (2015). Treatment to prevent HIV transmission in serodiscordant couples in Henan, China, 2006 to 2012. Clin Infect Dis.

[CR24] Sturmer M, Preiser W, Gute P, Nisius G, Doerr HW (2004). Phylogenetic analysis of HIV-1 transmission: pol gene sequences are insufficient to clarify true relationships between patient isolates. AIDS.

[CR25] Tamura K, Peterson D, Peterson N, Stecher G, Nei M, Kumar S (2011). MEGA5: molecular evolutionary genetics analysis using maximum likelihood, evolutionary distance, and maximum parsimony methods. Mol Biol Evol.

[CR26] Tang Z, Lan G, Chen YQ, Zhu Q, Yang X, Shen Z, Chen Y, Zhang H, Kan W, Xing H, Ruan Y, Shao Y (2015). HIV-1 treatment-as-prevention: a cohort study analysis of serodiscordant couples in rural Southwest China. Medicine.

[CR27] Trask SA, Derdeyn CA, Fideli U, Chen Y, Meleth S, Kasolo F, Musonda R, Hunter E, Gao F, Allen S, Hahn BH (2002). Molecular epidemiology of human immunodeficiency virus type 1 transmission in a heterosexual cohort of discordant couples in Zambia. J Virol.

[CR28] Wang N, Wei H, Xiong R, Zhang H, Ning C, Zhang L, Wang J, Feng Y, Shao Y (2013). Near full-length genome characterization of a new CRF01_AE/CRF08_BC recombinant transmitted between a heterosexual couple in Guangxi, China. AIDS Res Hum Retroviruses.

[CR29] Yang J, Ge M, Pan XM (2012). A time lag insensitive approach for estimating HIV-1 transmission direction. AIDS.

[CR30] Zeng H, Sun Z, Liang S, Li L, Jiang Y, Liu W, Sun B, Li J, Yang R (2012). Emergence of a new HIV type 1 CRF01_AE variant in Guangxi, Southern China. AIDS Res Hum Retroviruses.

