# Supplementary figures and images for: Molecular linkage tracing of HIV-1 transmission events in seroconcordant couples in Guangxi Province, Southeastern China
Source: Springerplus. 2016 Nov 25;5(1):2015. doi: 10.1186/s40064-016-3578-2 (PMC5124024; doi:10.1186/s40064-016-3578-2)

Supple 1

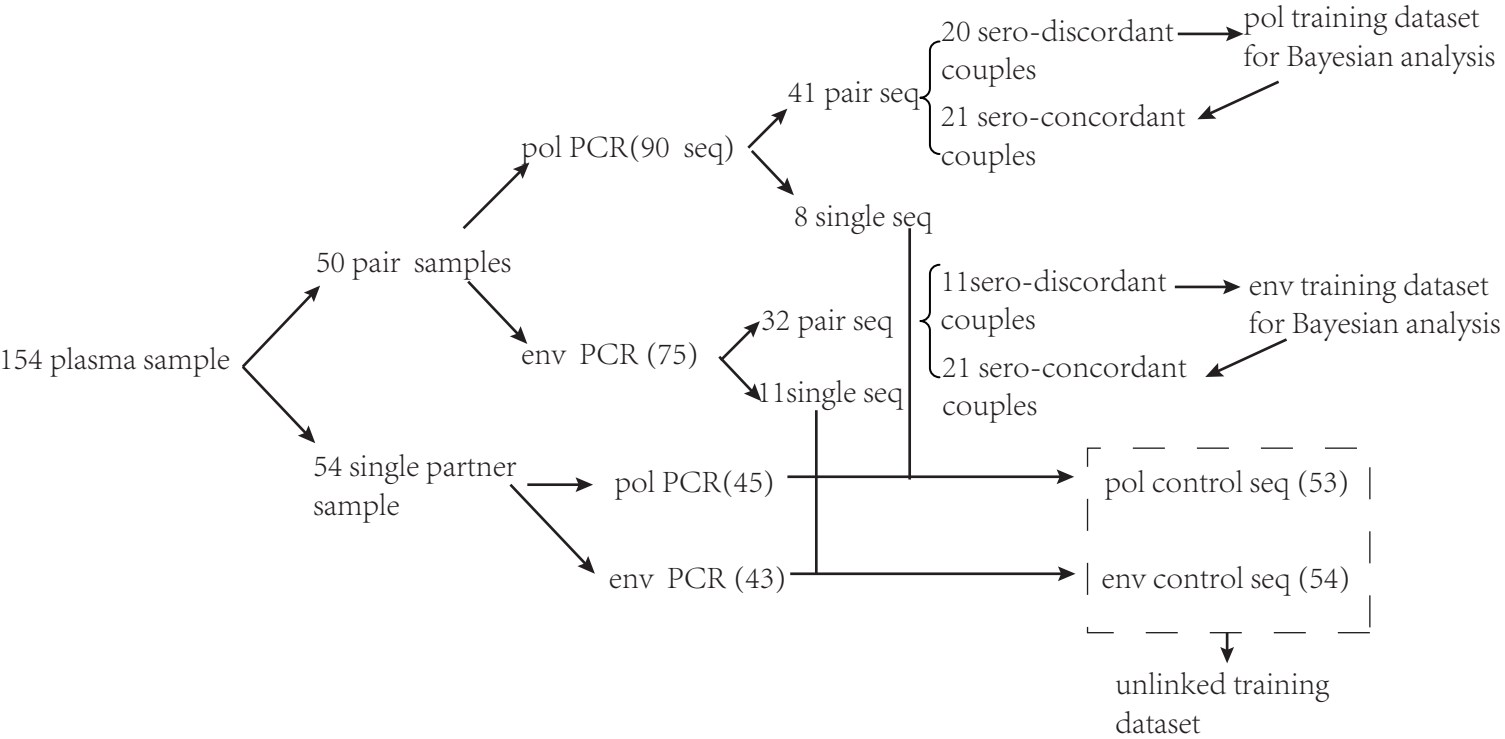

Supplement: Supplementary file 1 — Additional file 1: Figure S1. Flow chart depicting the process of screening our sample set and 339 determining genetic linkage and direction of transmission. [file 40064_2016_3578_MOESM1_ESM.pdf]
